# The effect of trauma and patient related factors on radial head fractures and associated injuries in 440 patients

**DOI:** 10.1186/s12891-015-0603-5

**Published:** 2015-06-05

**Authors:** Izaäk F. Kodde, Laurens Kaas, Nick van Es, Paul G.H. Mulder, C. Niek van Dijk, Denise Eygendaal

**Affiliations:** Department of Orthopedic Surgery, Upper Limb Unit, Amphia Hospital, Breda, The Netherlands; Department of Orthopedic Surgery, Academic Medical Center, Post-box 22660, 1100 DD Amsterdam, The Netherlands; Department of Orthopedic Surgery, University Medical Center Utrecht, Utrecht, The Netherlands; Consulting Biostatistician, Amphia Academy, Amphia Hospital, Breda, The Netherlands

**Keywords:** Associated injury, Elbow, Mechanism of injury, Radial head fracture, Trauma

## Abstract

**Background:**

Radial head fractures are commonly interpreted as isolated injuries, and it is assumed that the energy transferred during trauma has its influence on the risk on associated ipsilateral upper limb injuries. However, relationships between Mason classification, mechanism of injury, and associated injuries have been reported only once before in a relatively small population. The purpose of this study was to define whether trauma mechanism and patient related factors are of influence on the type of radial head fracture and associated injuries to the ipsilateral upper limb in 440 patients.

**Methods:**

The radiographs and medical records of 440 patients that presented with a fracture of the radial head were retrospectively analyzed. The medical records of all patients were searched for (1) the trauma mechanism and (2) associated injuries of the ipsilateral upper limb. The mechanism of injury was classified as being low-energy trauma (LET) or high-energy trauma (HET).

**Results:**

Associated injuries to the ipsilateral upper limb were present in 46 patients (11 %). The mean age of patients with associated injuries (52 years) was significantly higher compared to patients without associated injuries (47 years) (P = 0.038), and female patients with a radial head fracture were older than males. Injury patterns were classified as LET in 266 patients (60 %) and as HET in 174 patients. HETs were significantly more common in young men. Associated injuries were not significantly different distributed between HET versus LET (P = 0.82).

**Conclusions:**

Injuries concomitant to radial head fractures were present in 11 % of patients and the risk for these associated injuries increases with age. Trauma mechanism did not have a significant influence on the risk of associated injuries. Complex elbow trauma in patients with a radial head fracture seems therefore to be suspected based on patient characteristics, rather than mechanism of injury.

## Background

Radial head fractures are common with an estimated incidence of 28–39 per 100.000 inhabitants per year [1, 2]. The trauma mechanism of radial head and neck fractures is by indirect impact along the radius, usually caused by a fall on the outstretched hand in pronation and elbow in slight flexion [3, 2]. Previous epidemiological studies by Duckworth *et al.* and Kaas *et al.* revealed a mean age of 43 – 48 years and a significantly higher age of female patients compared to males. Both authors questioned whether this phenomenon could be caused by osteoporosis [2, 1]. In a subsequent case–control study including peripheral bone mineral density (BMD) measurements, radial head fractures in female patient above 50 years old were defined as potentially osteoporotic fractures [4].

Although radial head fractures are commonly seen as isolated injuries, associated injuries are reported in up to 92 % of cases [5]. More complex injuries according to the Mason classification seem to be associated with other associated ipsilateral upper limb injuries [2]. Both the complexity of the fracture pattern as per the Mason classification and the risk of associated ipsilateral upper limb injuries are assumed to be related to the energy transferred during trauma. However, little is known about the effects of patient and trauma related factors on the complexity of the radial head fracture and associated injuries.

Since significant symptomatic associated injuries are present in only a minority of radial head fractures, it might be helpful for the clinician to increase the à priory suspicion for more complex elbow trauma based on patient and injury characteristics. The purpose of this study was therefore to define whether trauma mechanism and patient related factors are of influence on the type of radial head fracture and associated injuries to the ipsilateral upper limb. We hypothesized that associated injuries are more common in the elderly and patients that sustained a high-energy trauma. In order to answer these study questions, the current report describes the assessment and analysis of 440 patients with a fracture of the radial head.

## Methods

Consecutive patients who presented with a fracture of the radial head at the emergency department (ED) of our hospital during a 4-year period were retrospectively identified. This level 2 trauma center provides a region of 400,000 inhabitants with acute medical care and is annually visited by approximately 44,000 patients. Trained residents in general and/or orthopedic surgery performed primary assessment and management of all patients at the ED. A trauma surgeon and an orthopedic surgeon reviewed the diagnosis and treatment. Inclusion criteria were radiographically confirmed acute fracture of the radial head and skeletal maturity. Patients with missing data about the mechanism of injury in their medical record or absence of initial radiographs were excluded. Approval for this study was waived by the ethics committee of the Amphia Hospital.

Radial head fractures on initial radiographs of the elbow obtained at the ED were classified according to the Mason classification [6]. The medical records (*e.g.* emergency notes) of all patients were searched for (1) the trauma mechanism and (2) diagnosis of associated injuries of the ipsilateral upper limb. Data extraction was performed by two of the authors. All radiographs of the ipsilateral upper limb and medical records were reviewed to identify possible associated injuries. Standardized radiographs with anteroposterior and lateral views were made in all patients. An additional radial head view was made in 120 patients and a CT-scan of the elbow in 30 patients. Additional imaging studies were not performed on a regular basis, but only if clinical assessment (*e.g.* physical examination) or primary radiographs indicated the probability of associated injuries. Retrospective assessment of radiographs was done by a senior orthopedic resident specially trained in radial head fractures. The mechanism of injury was noted as being low-energy trauma (LET) or high-energy trauma (HET), see Table 1 [2].

**Table 1:** Classification of mechanism of injury

Trauma mechanism	Examples
LET	Fall from standing position, fall from chair, fall during walking.
HET	Fall from roof, motor vehicle accidents, fall from bicycle or horse with speed, contact sports trauma.

Statistical analysis (SPSS 20.0 IBM corporation, Armonk, NY, USA) was performed using the independent *T*-test and one-way ANOVA test to compare numerical data between groups of patients and the Fisher’s exact test and Chi-squared test for categorical data. Odds ratios were obtained by logistic regression. In order to provide mutual independency between cases, only one of the cases with bilateral fractures was included in analyzes between groups. Results were considered statistically significant at P < 0.05.

## Results

**Fig. 1:**
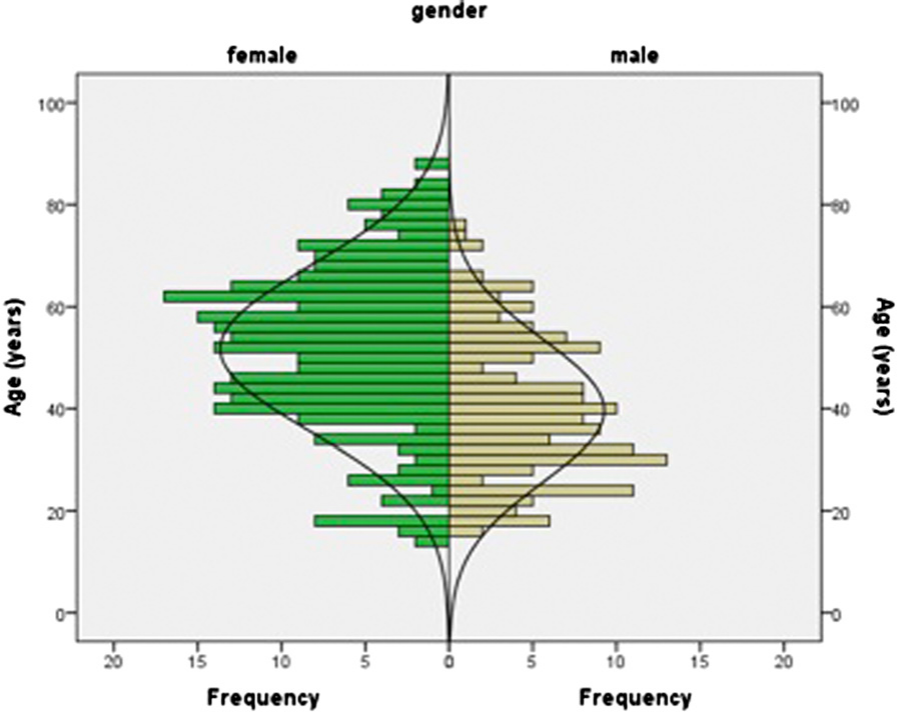
Age distribution of patients with a radial head fracture

**Fig. 2:**
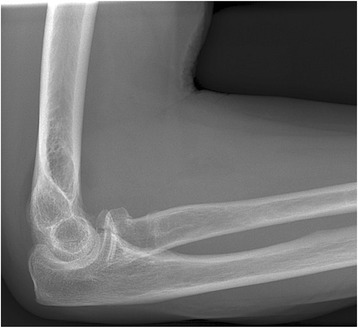
associated injury (coronoid fracture) on radiograph

Over the selected period, the records of 450 patients that presented with a radial head fracture were reviewed. Ten patients were excluded since there was no information about the mechanism of injury reported. The mean and median age of the remaining 440 patients was 47 years (range 14 – 88, SD 16.5), and there were 278 (63 %) female patients. The mean age was 40 years for male patients, which was significant younger than the 52 years for female patients (P < 0.001) (Fig. 1). Mason type-1 fractures were identified in 319 patients (73 %), type-2 fractures were seen in 84 patients (19 %) and type-3 fractures in 37 patients (8 %). Associated injuries to the ipsilateral upper limb were present in 46 patients (11 %) and are summarized in Table 2 and an example of an associated injury (coronoid fracture) on radiographs in Fig. 2. Injury patterns were classified as LET in 266 patients (60 %) and as HET in 174 patients. Ten patients (2 %) had a radial head fracture following elbow dislocation. Thirteen patients (3 %) sustained two radial head fractures during the inclusion period. Since mutual independency between cases was required, only one fracture per patient was included in statistical analyzes between groups.

**Table 2:** Types and frequencies of associated injuries

Types of associated injuries.	Mechanism of injury		Total (%)	Cumulative percent
	LET	HET		
Distal radius fracture	4	0	4 (8.7)	8,7
Carpal fracture	9	3	12 (26.1)	34,8
SL dissociation	1	1	2 (4.3)	39,1
Proximal ulna fracture	3	1	4 (8.7)	47,8
Coronoid fracture	7	7	14 (30.4)	78,3
UCL injury	0	1	1 (2.2)	80,4
LCL injury	1	1	2 (4.3)	84,8
Coronoid + LCL injury (dislocation)	0	3	3 (6.5)	91,3
Capitellum injury	1	1	2 (4.3)	95,7
DRUJ dislocation (Essex Lopresti injury)	1	0	1 (2.2)	97,8
Humeral fracture	0	1	1 (2.2)	100,0
Total	27	19	46

The mean age of patients with associated injuries (52 years) was significantly higher compared to patients without associated injuries (47 years) (P = 0.038). The odds for an associated injury for each year increase in age in females was 1.031 (95 % CI: 1.006-1.057; P = 0.015) and in males 0.989 (95 % CI: 0.950-1.029; P = 0.59). These odds ratios did not significantly differ between females and males (P = 0.082). The age-adjusted odds ratio of females to males was 1.189 (95 % CI: 0.532-2.660; P = 0.67).

Table 3 illustrates the distribution of Mason fracture types for associated injuries. There was a significant difference in Mason fracture type comparing patients with and without associated injuries (P < 0.001). Associated injuries were not significantly different distributed between HET versus LET (P = 0.82) (Table 4).

**Table 3:** Distribution of Mason fracture types for associated fractures

		Associated injury		Total
		-	+	
Mason type	1	289	19	308
	2	72	10	82
	3	20	17	37
Total		381	46	427

**Table 4:** Distribution of HET versus LET for associated injuries

		Associated injury		Total
		-	+	
Mechanism of injury	LET	230	27	257
	HET	151	19	170
Total		381	46	427

Mean age of patients with a HET was 43 years compared to 51 years for LET (P < 0.001). The distribution of HET versus LET was significantly different for males versus females, with more HET for male patients (P < 0.001). There was no significant difference in Mason fracture type for HET versus LET. There were no age-specific differences for the type of Mason fracture (P = 0.23).

## Discussion

The radial head is an important secondary stabilizer of the elbow [7]. Especially in combination with deficiency of the collateral ligaments or coronoid fractures, elbow stability heavily relies on an intact radial head [8]. Accurate recognition and assessment of all associated injuries are necessary to initiate the correct treatment of the injury; early adequate management is mandatory to improve the clinical outcome [8, 9]. The radial head fracture is, in general, believed to be an isolated, simple injury with a benign outcome. However, associated injuries with radial head fractures are increasingly recognized for their clinical significance [10–12]. The current study revealed an 11 % risk of symptomatic associated injuries, which increases with age and Mason fracture type.

It is thought that the complexity of the injury is related to the amount of energy transferred during the trauma. However, in this study energy transfer during trauma was not related to associated injuries or Mason fracture type. Radial head fractures following HET occurred most frequently in young men. HET did not lead to more associated injuries or more complex radial head fractures than LET in the current study. The only predictors for more associated injuries were a higher Mason fracture type and a higher age. High-energy transfers through the elbow joint during trauma did not result in more concomitant injuries in young patients, in contrast to older patients that sustain LET more commonly. Based on these observations, we assume that additional injuries, next to radial head fractures, were also dependent on the intrinsic quality of bone and soft tissues and a frailty phenotype of the elderly patient. Therefore, complex elbow trauma in patients with a radial head fracture should be suspected based on patient characteristics rather than the mechanism of injury.

Mechanism of injury of radial head and neck fractures have been categorized by Duckworth *et al.* in low-energy (*i.e.* fall from standing height) and high-energy (*e.g.* sports trauma or fall from height), as is done in the current study [2]. They observed significant more HETs in males, and HET was defined as a significant risk factor for associated injuries. HETs occurred in 43 % of the 285 patients in their series and associated injuries were present in 7 % of patients. The current study identified 40 % of injuries as HET, and 11 % of patients had associated injuries. However, HETs were no significant risk factors for additional injuries in this series. One explanation for this difference might be that Duckworth analyzed both radial head and neck fractures, whereas we included only radial head fractures. They suggested that radial neck fractures were – more than radial head fractures – low-energy fragility fractures. Another explanation might be that associated injuries occur by the direction of impact during trauma rather than by the amount of energy transferred during trauma. McGinley *et al.* found that axial impact in neutral position of the forearm resulted in isolated radial head fractures, whereas loading in pronation resulted in more comminuted fractures with associated lesions of the interosseous membrane [13]. Thus, *how* the energy is transferred may be more important than *how much* energy is transferred during trauma.

In this study, associated injuries were more frequently observed in the elderly, and female patients with a radial head fracture were older than males. These findings are in agreement with suggestions that radial head fractures are potentially osteoporotic fractures [4]. Gebauer *et al.* recently performed cadaveric studies on the microarchitecture of the radial head. BMD and histomorphometric analysis were performed on radial heads of three different groups of cadavers; aged 20–40 years, 41–60 years, and 61–80 years. They showed a significant decrease in BMD with increasing age for men and women. Trabecular thickness significantly decreased with age, whereas trabecular separation significantly increased with age, leading to more radiolucency of the radial head on radiographs with aging. These age-related changes in microarchitecture of the radial head were suggested to reduce the biomechanical stability of the bone [14]. Since associated injuries were more commonly observed in older patients in the current study, we assume that older female patients with a radial head fracture overall have a diminished bone quality. As the extent of the injury varies with the position of the forearm during a fall, the way elderly fell may also account for the higher amount of associated injuries. Overall, a frailty phenotype is associated with a greater risk of fracture, disability and falls in elderly women [15]. It seems therefore critical to maintain optimal bone and soft tissues qualities, as well as overall physical condition, in the aging population in order to prevent more complex elbow injuries.

The more complex the radial head fracture is according to the Mason classification, the higher the frequency of associated injuries in the current study. Associated injuries were identified in almost half of the patients with a Mason type-3 fracture of the radial head. Kaas *et al.* noticed associated injuries on MRI in even 100 % of patients with a Mason type-3 fracture [16]. Therefore, thorough evaluation of the elbow by a preoperative CT- or MRI-scan and/or intraoperative stability testing using fluoroscopy might be preferable to adequately deal with possible associated injuries in all Mason type-3 fractures. With regard to Mason type-2 fractures, Rineer *et al.* identified concomitant injuries in 77 % of patients [17]. They divided Mason type-2 fractures in two groups based on the existence of cortical contact between the fracture fragment and the radial head. Fracture fragments without cortical contact led to a 21-times greater risk for associated injuries. Loss of cortical contact was in the current study seen in 37 % (17/46) of patients with associated injuries, versus 6 % (24/381) of patients without associated injuries. However, the distribution of radial head fractures among the Mason fracture types in the study from Rineer [17] is variant to those of Kaas [1], Duckworth [2], and the current study, as is the amount of associated injuries for Mason type-2 fractures. Nevertheless, as Rineer *et al.* concluded: ‘if imaging studies fail to demonstrate the presence of a complex elbow injury pattern, the surgeon should still have a high suspicion that some degree of occult instability may be present’ in Mason type-3 fractures and Mason type-2 without cortical contact [17].

The current study was with 440 included patients one of the largest studies on the epidemiology of radial head fractures [2, 1, 18, 19]. The influences of mechanism of injury on radial head and neck fractures and associated injuries have only been once reported before [2]. The current study focused both on modes of injury and patient related factors on associated injuries for radial head fractures, leading to other findings. This study had several limitations in addition to its retrospective nature. First, associated injuries were documented based on available imaging studies instead of standardized radiographic studies for all patients. For instance, a standard MRI was not performed, and the LCL lesions in this study were identified as avulsion fractures from the epicondyle or during surgical reconstruction of Mason type 3 fractures. However, since most associated injuries are not clinically relevant, it is not advised to perform MRI scans on a regular basis for radial head fractures [10]. Second, the interobserver reliability of the Mason classification is known to be only moderate [20]. In addition, it might be difficult to take standardized radiographs of the elbow in the acute setting because of pain. Third, there is no clear definition of amounts of energy that are transferred during different mechanisms of injury. Moreover, the direction of impact (axial, direct, rotation of forearm, *etc.*) during trauma, may be more important to sustain associated injuries than the kind of trauma [21].

Future research should concentrate on Mason type-2 and type-3 fractures and the association with clinically and therapeutically relevant associated injuries. The impact of energy transfer during trauma and its consequences on associated injuries should be established. The indications for additional imaging studies for these fractures need to be explored.

## Conclusions

Injuries concomitant to radial head fractures were present in 11 % of patients and the risk for these associated injuries increases with age. Furthermore, the risk for associated injuries increases with complexity of the radial head fracture according to the Mason classification. Trauma mechanism did not have a significant influence on the risk of associated injuries. Complex elbow trauma in patients with a radial head fracture seems therefore to be suspected based on patient characteristics, rather than mechanism of injury. Associated injuries should be actively be explored in older patients with Mason type-2 or type-3 fractures by means of physical examination of the whole ipsilateral arm and potential additional radiographic studies.

## Abbreviations

(BMD): Bone Mineral Density
(CI): Confidence Interval
(ED): Emergency Department
(HET): High-Energy Trauma
(LET): Low-Energy Trauma
(MRI): Magnetic Resonance Imaging
(SD): Standard Deviation
